# Establishment of a One–Pot RAA–CRISPR/Cas13a Assay-Based TGEV S Gene Detection

**DOI:** 10.3390/vetsci12050464

**Published:** 2025-05-12

**Authors:** Lindan Lv, Hao Mu, Shaomei Li, Jieqi Gao, Mingni Liu, Shuizhu Niu, Guoyang Xu, Lizhi Fu, Zhenhui Song, Liu Yang

**Affiliations:** 1 National Center of Technology Innovation for Pigs, Chongqing 402460, China; lvlindan@foxmail.com (L.L.); mouh@cqaa.cn (H.M.); shaomeili123@163.com (S.L.); 15035607783@163.com (J.G.); declanlmnyui@163.com (M.L.); cecilianiu@outlook.com (S.N.); guoyangxu@126.com (G.X.); fulz@cqaa.cn (L.F.); 2 College of Veterinary Medicine, Southwest University, Chongqing 400715, China; 3 Chongqing Academy of Animal Sciences, Chongqing 402460, China; 4 Chongqing Research Center of Veterinary Biologicals Engineering and Technology, Chongqing 400715, China

**Keywords:** TGEV, RAA, CRISPR/Cas13a, detection method

## Abstract

Porcine transmissible gastroenteritis virus (TGEV) is a highly contagious pathogen causing severe diarrhea in pigs, particularly piglets, leading to significant economic losses. Distinguishing TGEV from the genetically similar porcine respiratory coronavirus (PRCV) remains challenging due to their high genomic homology. In this study, we developed a one–pot assay combining recombinase-aided amplification (RAA) and CRISPR/Cas13a technology, targeting the TGEV S gene. This method was optimized for sensitivity and specificity, with orthogonal tests determining the optimal reagent concentrations. The assay achieved a detection limit of 4.13 copies/µL within 40 min at 37 °C, demonstrating no cross-reactivity with other porcine viruses. Clinical validation on 140 samples showed 100% concordance with RT–qPCR and RT–PCR results. Since the established method is completed in a single reaction tube, it eliminates the need for step-by-step operations, simplifying the process and reducing the risk of cross–contamination and false positives in subsequent tests. Overall, this assay shows promising potential for TGEV detection.

## Simple Summary

Porcine transmissible gastroenteritis virus (TGEV) is a major cause of severe diarrhea in pigs, leading to high mortality and significant economic losses. Due to the high genomic similarity between TGEV and porcine respiratory coronavirus (PRCV), it is difficult to distinguish them serologically. In this study, we developed a specific, rapid, and sensitive nucleic acid detection method for TGEV using a combination of recombinase–aided amplification (RAA) and the CRISPR/Cas13a system. This one–pot assay, performed at 37 °C, can detect TGEV within 40 min, with a sensitivity of 4.13 copies/µL. When tested on 140 clinical samples, the method demonstrated 100% accuracy compared with RT–qPCR and RT–PCR. Its simplicity and efficiency make it a promising tool for timely detection and control of TGEV infection.

## 1. Introduction

Porcine transmissible gastroenteritis virus (TGEV) is a highly contagious coronavirus that causes severe diarrhea, vomiting, and dehydration in pigs. In piglets under two weeks of age, mortality rates can reach 100%, while surviving animals exhibit stunted growth, significantly reducing farm productivity and leading to significant economic losses in the swine industry due to high mortality, reduced growth rates, and costly containment measures [1,2,3]. There are no specific drugs or effective treatments for TGE, except for immunization. However, vaccines do not provide immune protection against TGEV variants. Therefore, timely detection and accurate diagnosis of TGEV infection are crucial for the scientific and effective prevention and control of the disease [4]. Traditional methods for TGEV diagnosis include virus isolation and identification, the ELISA, and PCR, among others. However, virus isolation is time-consuming, typically taking 3–7 days, and requires specialized biosecurity facilities. ELISA offers moderate sensitivity, with a detection limit of approximately 10^3^ TCID_50_/mL; its utility is limited by the high genetic and antigenic similarity between TGEV and PRCV [5,6,7], a TGEV S gene deletion mutant strain that shares up to 96% nucleic acid identity at the genomic level [8,9]. This makes it challenging to distinguish between the two viruses using serological methods. Although PCR and real–time PCR provide high sensitivity, with detection limits as low as 10^1^–10^2^ copies/μL, these methods mainly target conserved regions such as the M or N genes, making it difficult to distinguish between TGEV and PRCV in clinical settings.

In recent years, clustered regularly interspaced short palindromic repeat (CRISPR)/CRISPR–associated protein (Cas) systems have attracted significant scientific attention. Among them, the CRISPR/Cas13a detection platform has emerged as a powerful tool for molecular diagnostics, enabling the selective activation of Cas13a nuclease activity through guide RNA–mediated targeting of specific nucleic acids [10,11,12]. This technology has been successfully applied to detect various pathogens, including African swine fever virus (ASFV) [13], porcine epidemic diarrhea virus (PEDV) [14], and severe acute respiratory syndrome coronavirus 2 (SARS-CoV-2) [15]. Meanwhile, recombinase–aided amplification (RAA) has gained recognition as an advanced nucleic acid amplification technique. With its remarkable temperature adaptability, rapid reaction time, and simple operation, RAA is particularly suitable for diverse detection environments. Unlike conventional PCR methods, which require thermal cycling (denaturation, annealing, and extension), RAA achieves efficient amplification under isothermal conditions, making it highly valuable for agricultural pathogen detection [16,17].

In this study, we designed specific RAA primers and ssRNA reporter probes and developed a simple, specific, and sensitive novel detection system targeting the TGEV S gene by combining CRISPR/Cas13a technology with RAA. This integrated approach overcomes the limitations of traditional detection methods and is expected to provide valuable technical support for the early detection of TGEV.

## 2. Materials and Methods

### 2.1. Viruses and Samples

Porcine delta coronavirus (PDCoV), porcine reproductive and respiratory syndrome virus (PRRSV), porcine circovirus 2 type (PCV2), and porcine kobuvirus (PKoV) were previously isolated and stored in the National Center of Technology Innovation for Pigs. TGEV (strain SCJY–1), porcine epidemic diarrhea virus (PEDV) (strain SCSZ–1), and classical swine fever virus (CSFV) were isolated from vaccines produced by Chongqing Aolong Biological Products Co., Ltd. PRCV was kindly provided by the College of Animal Medicine, Southwest University. A total of 140 samples (including feces, intestinal tissues, and swabs) from porcine diarrhea cases were collected across different geographical locations, primarily from pig farms in the Luzhou, Dazhou, Longchang, Wulong, Changshou, Wanzhou, Banan, and Rongchang districts. All samples were stored at the National Animal Disease–Chongqing Monitoring Station and the Chongqing Academy of Animal Sciences.

### 2.2. Nucleic Acid Extraction

Viral RNA/DNA was extracted using the TaKaRa MiniBEST universal RNA/DNA extraction kit (TaKaRa, Dalian, China), following the manufacturer’s instructions. The concentration of the extracted RNA was quantified using spectrophotometry, with A260/A280 ratios between 1.8 and 2.0 confirming RNA purity. The reverse–transcription reaction was performed using the PrimeScript™ RT Reagent Kit (TaKaRa, Dalian, China) to synthesize cDNA from the extracted RNA. The extracted nucleic acid and cDNA samples were stored at −80 °C.

### 2.3. Construction of Standard Recombinant Plasmid Containing Target Sequence

Since PRCV is a variant of TGEV, the whole genome sequences of TGEV and PRCV were downloaded from the GenBank database (http://www.ncbi.nlm.nih.gov/, accessed on 27 May 2024). Sequence alignment was carried out using the bioinformatic analysis software application MEGA 7.0, and DNA sequences present in the TGEV S gene but absent in PRCV were selected for subsequent studies. One of the consensus sequences with 100% alignment with the S gene was synthesized as the target sequence and cloned into a pUC57 vector (Sangon Biotech, Shanghai, China) to construct recombinant plasmids, named pUC57–TGEV. The plasmids were transformed into *E. coli* DH5α for cultivation. Positive plasmids were extracted using the E.Z.N.A Plasmid Mini–Extraction Kit (Omega Biotek Inc., Shanghai, China) and sequenced by Sangon Biotech Co., Ltd. The sequencing results confirmed the presence of the TGEV sequence. The recombinant plasmid was used as a standard positive control and stored at −20 °C for future use. The concentration of the extracted recombinant plasmid was determined using an ultraviolet spectrophotometer (OD260) (Thermo Fisher, Waltham, MA, USA), and the DNA copy number was computed as follows: dsDNA copy number (copies/μL) = (6.02 × 10^23^ (copies/mol) × concentration (ng/μL) × 10^−9^)/(DNA length × 660) [18,19].

### 2.4. Quantitative Real–Time PCR (qPCR) Assay

According to the requirements of the qPCR assay, one set of primers (F:5′-AGTCGTTAATGGATACCCA-3′, R: 5′-TGCACTCACTACCCCAATT-3′) and a probe (FAM-TCTGCTGAAGGTGCTATTAT-TAMRA) were designed from the conserved nucleotide region of the target sequence using Primer Premier 5.0 software (PREMIER Biosoft, San Francisco, CA, USA). The reaction system was 25 μL in volume, which included 12.5 μL of 2 × probe qPCR Mix with UNG (TaKaRa, Beijing, China), 0.5 μL of each F/R primer (10 μM), 1 μL of the probe, 2 μL of sample DNA/cDNA, and 8.5 μL of ddH_2_O. All qPCR assays were carried out using a CFX96 Real-Time System (Bio-Rad, Shanghai, China) according to the following procedures: UNG action at 25 °C for 10 min, pre–denaturation at 95 °C for 30 s, 40 cycles of denaturation at 95 °C for 5 s, and annealing at 54 °C for 30 s, with the FAM fluorescent signal detected every 30 s. A series of 10–fold consecutive dilutions of the pUC57–TGEV plasmid were used as a template for the qPCR assay to construct the standard curves, which were generated based on the cycle threshold (Ct) values and the copy numbers (Log values) of the template DNA. The coefficients of determination (R^2^) were calculated using the software application GraphPad Prism, v.8.0.1 (https://www.graphpad.com/scientific-software/prism/, accessed on 27 May 2024). The experiment was also performed three times independently to verify its repeatability. We evaluated the method’s detection capability by obtaining 10–fold serial consecutive dilutions of the pUC57–TGEV plasmid as the template for the qPCR assay sensitivity test, and the detection limit of the latter was used as a template to conduct three duplicate tests with three duplicate wells, each for the determination of the repeatability of this method. Lastly, seven common porcine–derived nucleic acids of viruses, including TGEV, PRCV, PCV2, PKoV, PEDV, PRRSV, and PDCoV, were selected as control templates for the assessment of the detection specificity of our method.

### 2.5. RAA Primers, ssRNA Reporter, and crRNA Preparation

Five RAA primers were designed online (https://ezassay.com/primer, accessed on 27 May 2024) based on the conserved nucleotide region of the TGEV S gene, according to the design requirements for RAA primers, which were synthesized by Sangon Biotech (Shanghai, China). A total of 2 forward primers and 3 reverse primers were paired, resulting in 6 pairs of primers, to determine the optimal primer combinations (Table 1). Basal RAA was performed with 10^4^ copies/μL of the recombinant plasmid (pUC57–TGEV) as the template, according to the instructions provided with the RAA Nucleic Acid Amplification Kit (Zhuangbo, Guangxi, China). The best primer pair was identified by analyzing the brightness of the gel electrophoresis bands of 7 μL RAA products using the Image J 1.54 analysis tool. ddH_2_O was used as a negative control.

The crRNA (5′-GAUUUAGACUACCCCAAAAACGAAGGGGACUAAAACUCAGCAGAAU UAAAAUUGCGGGUUGUUG-3′) was designed and synthesized to target the RAA amplification product, according to the instructions for the enzyme LwaCas13a (Novoprotein, Suzhou, China) to ensure the proper functioning of the CRISPR/Cas13a reactive system; the 3′–end of the crRNA was ligated with palindromic repeat sequences (underlined) that bind to the Cas13a enzyme, forming a binary complex. Due to the cascade shearing activity of the Cas13a enzyme on RNA, the 5′- and 3′–ends of the ssRNA reporter were labeled with fluorescein FAM and quenching BHQ1 motifs, respectively, obtaining the sequence 5′-FAM–rUrUrUrUrUrU–BHQ1-3′. The RAA primers and crRNA were evaluated using a nucleotide BLAST + 2.15.0 search in the National Center for Biotechnology Information (NCBI) database, revealing no matches with sequences from other species.

### 2.6. CRISPR/Cas13a Complex Cleavage System

A cleavage premix system was prepared according to the CRISPR/Cas13a manual to verify the Cas13a cleavage activity and the accuracy of the crRNA. The 20.0 μL reaction system was composed of 2.0 µL of CRISPR/Cas13a 10 × buffer, 2.0 µL of LwaCas13a (200 pM), 8.0 µL of ddH_2_O, 2.0 µL of crRNA (20 μM), 1.0 µL of the ssRNA reporter (20 mΜ), and 5.0 µL of the RNA template. The master mix was quickly placed into a real–time PCR instrument, where FAM channel fluorescence values were collected every 15 s at 37 °C for 40 min. Three replicates were set up for each cleavage reaction to obtain mean Ct values and identify optimal amplification, and the TGEV RNA template was replaced with ddH_2_O as a negative control.

### 2.7. Establishment of TGEV Assay Based on RAA–CRISPR/Cas13a System

Based on the instructions of the fluorescein RAA nucleic acid amplification kit and the CRISPR /Cas13a manual, the RAA–CRISPR/Cas13a premix system for each reaction tube totaled 42.5 µL and consisted of 25.0 µL of A Buffer, 2.0 µL of the optimal forward primer with the T7 promoter (5′-GAAATTAATACGAC TCACTATAGGG-3′), 2.0 µL of the optimal reverse primer, 0.5 µL of LwaCas13a (5.0 pmol/µL), 0.25 μL of the crRNA (10.0 µM), 1.5 µL of T7 polymerase (50 U/µL; TaKaRa, Beijing, China), 2.0 µL of ribonucleotide triphosphate (rNTP) mix (50 mM, TaKaRa, Beijing, China), 2.0 µL of RNase inhibitor (40 U/µL; TaKaRa, Beijing, China), 0.5 μL of MgCl2 (500 mM), 0.5 µL of fluorescent ssRNA reporter (Bio–LifeSci, Shanghai, China), and 6.25 μL of RNase–free ddH_2_O. After thorough mixing, 5.0 μL of standard plasmids at a concentration of 104 copies/μL was added to the reaction tube as a template; then, 2.5 μL of B Buffer was added to the inner cap of the assay tube, the cap was quickly closed, and the contents were mixed upside down and briefly centrifuged. The master mix was then placed into a real–time PCR instrument, where FAM channel fluorescence values were collected every 30 s at 37 °C for 40 min. Three replicates were set up for each cleavage reaction to obtain the mean Ct values and verify optimal amplification. ddH_2_O was used as a negative control.

### 2.8. Optimization of TGEV Assay System Based on RAA–CRISPR/Cas13a

Given that RAA primer pairs, crRNA, and reporter RNA concentrations significantly influence the RAA–CRISPR/Cas13a reaction system, each element was classified into three levels, and the L9 (3^3^) orthogonal test scheme was designed for the reaction system (Table 2). This optimized protocol uses the optimal primer pairs for fluorescence detection every 30 s for a total of 80 cycles. Each trial was repeated three times to obtain the mean Ct values.

### 2.9. Specificity Analysis Test of RAA–CRISPR/Cas13a Assay

Nucleic acids from TGEV, PCV2, PDCoV, PKoV, PEDV, PRRSV, and PRCV were used as templates to perform the specificity analysis of the TGEV assay based on RAA–CRISPR/Cas13a, assessing cross–reactivity with other pig–derived viruses or genomes. ddH_2_O was used as a negative control.

### 2.10. Sensitivity Analysis Test of RAA–CRISPR/Cas13a Assay

The recombinant plasmid standard was consecutively diluted 10–fold with ddH_2_O until a concentration of 10^−2^ copies/μL was reached. The sensitivity of the RAA–CRISPR/Cas13a assay was validated using the recombinant plasmid at 10^3^–10^−2^ copies/μL as the standard nucleic acid template, with three replicates per reaction for each concentration to obtain the mean Ct values.

### 2.11. Stability Analysis Test of RAA–CRISPR/Cas13a Assay

Multiple replicates of the RAA–CRISPR/Cas13a assay were performed using the plasmid template at the detection limit for the evaluation of the stability of the detection method.

### 2.12. Effectiveness Evaluation of Testing Clinical Samples

A total of 140 clinical samples from diarrheic pigs were tested simultaneously to analyze the coincidence rate between the RAA–CRISPR/Cas13a assay, the RT–qPCR assay, and the RT–PCR assay for the further assessment of the reliability of the RAA–CRISPR/Cas13a assay in clinical detection.

### 2.13. Statistical Analysis

The statistical significance of the differences was determined using SPSSAU (https://spssau.com/index.html, accessed on 27 May 2024) to perform ANOVA and calculate the mean Ct values. Statistical analyses considered *p*-values < 0.05 as significant and *p*-values < 0.01 as highly statistically significant.

## 3. Results

### 3.1. Standard Plasmid Containing Target Sequence

**Figure 1:**
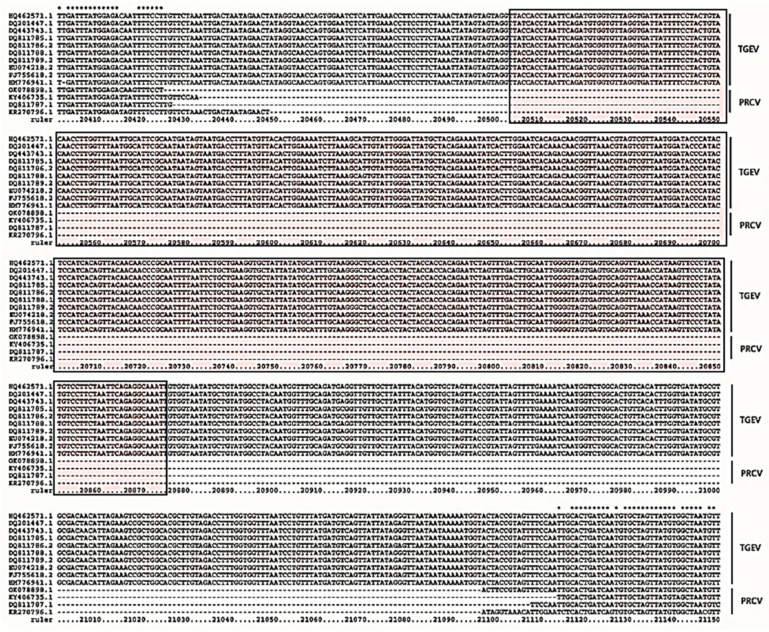
Partial sequence alignment of the S genes in different TGEV and PRCV strains; the consensus sequence within the box is the target sequence for detection. (Red mark) A 362 bp consensus nucleic acid sequence used for constructing the TGEV–puc57 recombinant plasmid.

The results of genome sequence alignment revealed significant differences in the S gene between TGEV and PRCV (Figure 1), consistent with previous reports [20,21]. A 362 bp consensus nucleic acid sequence (highlighted in the box), present in the S gene of TGEV but deleted in PRCV, was selected as the target for TGEV detection. This sequence was synthesized and ligated into the pUC57 vector, successfully constructing the recombinant plasmid pUC57–TGEV, whose sequencing results met expectations. The recombinant plasmid, extracted from positive clone bacteria, was measured to have a concentration of 139 ng/µL, corresponding to 4.13 × 10^10^ copies/µL.

### 3.2. Sensitivity and Specificity Validation of qPCR

**Figure 2:**
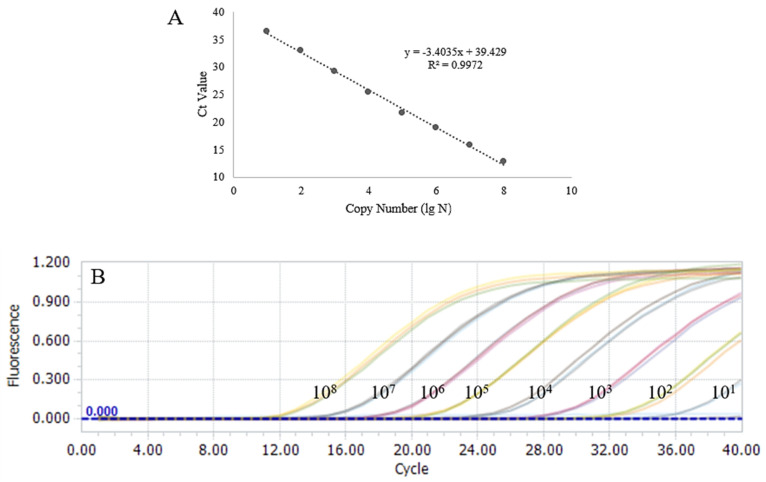
Sensitivity validation of qPCR. (**A**) Ten–fold serial dilutions of the TGEV plasmid were used as the template to construct the standard curve for qPCR. (**B**) Ten–fold serial dilutions of the pUC57–TGEV plasmid were used as the template for sensitivity detection in qPCR.

**Figure 3:**
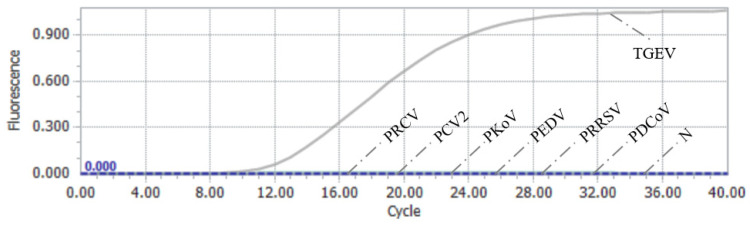
Specificity detection of qPCR.

A TaqMan qPCR method for detecting porcine TGEV was developed based on the screened target sequence of the S gene (sequence site: 20,517–20,879; GenBank ID: DQ811785). The detection limit of the qPCR method for the standard plasmid was 41.3 copies/μL (Table 3). The construction of the standard curves demonstrated a strong linear correlation (R^2^ = 0.9972, y = −3.4035lgN + 39.429) between the Ct values and the corresponding copy numbers of the target sequence in the TGEV S gene (Figure 2). Specificity evaluation revealed that the qPCR method produced an amplification curve exclusively for TGEV (Figure 3), and the coefficient of variation (CV) values for the nine detection tests at different concentrations of the standard plasmid were all below 1.5% (Table 3).

### 3.3. Optimal Primer Screening for RAA

**Figure 4:**
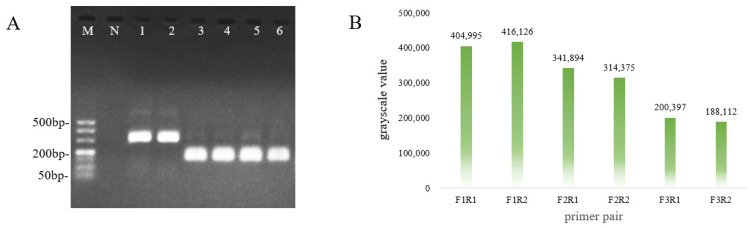
Determination of optimal primer pairs for the RAA method. (**A**) Optimal primer pairs were identified with the RAA method. (**B**) Image J analysis tool was used to evaluate the primer pairs and determine the most suitable one. M: DL500 DNA Marker; N: negative control; 1–6: primer pairs—F1/R1, F1/R2, F2/R1, F2/R2, F3/R1, and F3/R2, respectively.

Six primer pairs were tested using basal RAA with 4.13 × 10^4^ copies/μL of the pUC57–TGEV plasmid as the template to identify the optimal primer pairs for the method to ensure its specificity and effectiveness. The electrophoresis results showed that the sizes of the main bands of all RAA products were close to the theoretical values (Figure 4A), and the sequencing results met expectations. Only Lane 3 (F2/R1) and Lane 4 (F2/R2) displayed single bands with no non–specific bands. Further analysis of the gray values of the electrophoretic bands indicated that the band in Lane 3 was brighter than that in Lane 4 (Figure 4B). Based on these criteria, the optimal primer combination for RAA detection of TGEV is F2/R1.

### 3.4. Detection of CRISPR/Cas13a Complex Cleavage

**Figure 5:**
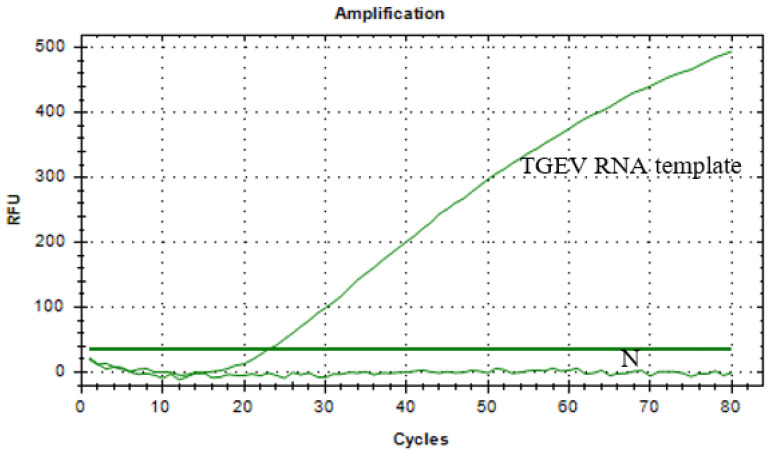
Detection results of the CRISPR/Cas13a complex cleavage system.

For the CRISPR/Cas13a cleavage premix system, fluorescence signal values were successfully detected using a real–time PCR instrument (Figure 5). The results indicated that the template was TGEV RNA, and the fluorescence signal values gradually increased over time, validating the Cas13a protein cleavage activity and accuracy of the crRNA. This indicates that the Cas13a protein binds to the palindromic sequence attached to the crRNA, forming a binary complex. Once the crRNA correctly pairs with the detected target fragment of TGEV RNA, the Cas13a protein triggers its shearing activity, cleaving the six–base ssRNA reporters in the system and releasing fluorescent signals that can be captured.

### 3.5. Orthogonal Test Optimization and Establishment of RAA–CRISPR/Cas13a Assay

The results of the L9 (3^3^) orthogonal tests, along with their analysis, are presented in Table 4 and Table 5. The k–values in Table 5 represent the average Ct values at different levels for each element, with a smaller k–value indicating higher detection efficiency and stability. Based on these reference conditions, the optimal combination is A3B2C3, suggesting that the optimal reaction conditions for efficiently amplifying TGEV nucleic acids are 12 μM ssRNA reporter, 10 μM crRNA, and 10 μM primer. The R values in Table 5 reflect the effect of each element on the RAA–CRISPR/Cas13a test, with larger R values indicating a greater influence of that element at its respective level. According to the R values from the L9 (3^3^) results, the influence of the three elements is ordered as follows: A (ssRNA reporter) > C (crRNA) > B (RAA primer). The results of the three–factor analysis of variance (ANOVA) showed the value as R^2^ = 0.962 (Table 6), indicating that the three factors—probe, primer, and crRNA—explain 99.6% of the variation in the Ct value, which means that the data from this experiment have statistical significance. Therefore, according to the above analysis, the optimized reaction system was established for the TGEV nucleic acid assay using the RAA–CRISPR/Cas13a method.

### 3.6. Results of Specificity Assessment of RAA–CRISPR/Cas13a Assay

**Figure 6:**
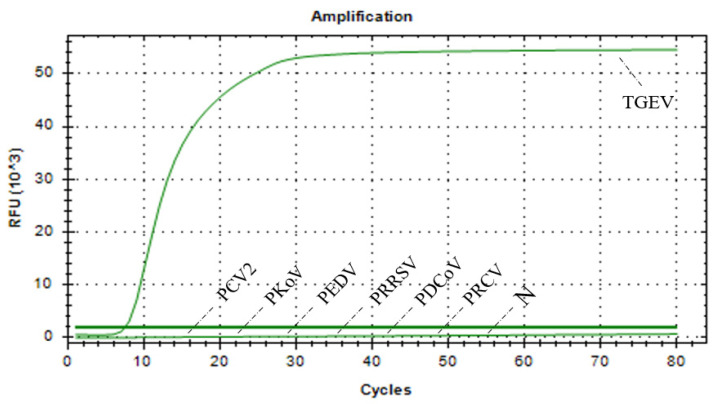
Results of the RAA–CRISPR/Cas13a specific test.

cDNA/DNA from TGEV, PCV2, PKoV, PEDV, PRRSV, PDCoV, and PRCV, all common pathogens that infect pigs, were used as nucleic acid templates for the assessment of the specificity of the RAA–CRISPR/Cas13a assay. As shown in Figure 6, the RAA–CRISPR/Cas13a assay did not produce a specific fluorescent signal for any pathogens other than TGEV, indicating that this method demonstrates high detection specificity for TGEV (Figure 6).

### 3.7. Results of Sensitivity Assessment of RAA–CRISPR/Cas13a Assay

**Figure 7:**
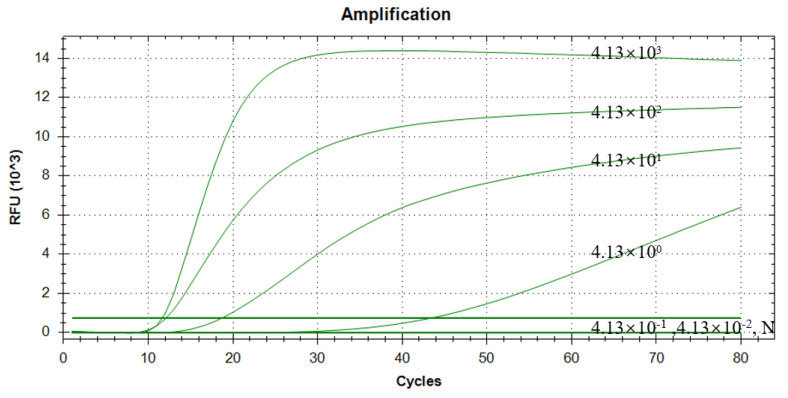
Sensitivity test results of plasmid templates with concentrations ranging from 4.13 × 10^3^ to 4.13 × 10^−2^ copies/µL, detected with RAA–CRISPR/Cas13a.

A series of 10–fold dilutions of the pUC57–TGEV recombinant plasmid (ranging from 4.13 × 10^3^ to 4.13 × 10^−2^ copies/µL) were tested as templates to investigate the sensitivity of the RAA–CRISPR/Cas13a assay. As shown in Figure 7, the results indicated that the minimum detectable copy number of pUC57–TGEV when using this method was 4.13 × 10^0^ copies/µL (95% CI: 29.47–32.11 Ct). In comparison, the detection limit of the previously established qPCR method was 4.13 × 10^1^ copies/µL, demonstrating that the RAA–CRISPR/Cas13a assay has higher detection sensitivity for TGEV.

### 3.8. Results of Stability Assessment of RAA–CRISPR/Cas13a Assay

**Figure 8:**
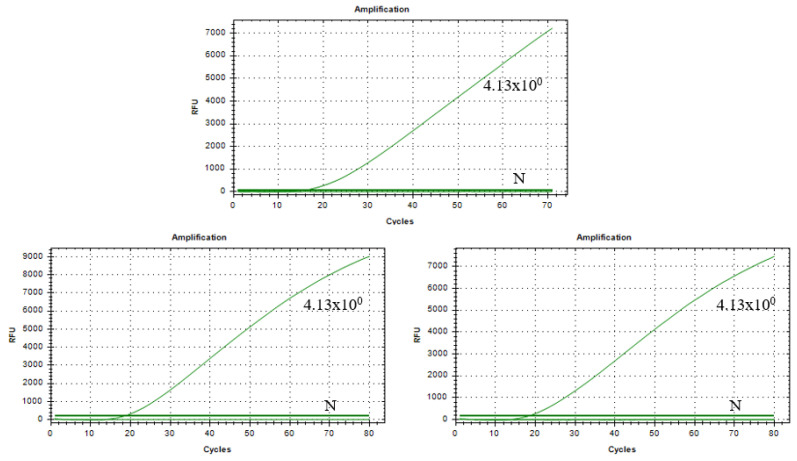
Results of the reproducibility test at a plasmid concentration of 4.13 × 10^0^ copies/µL.

For the plasmid templates with a detection limit of 4.13 × 10^0^ copies/µL, results from three replicate experiments consistently showed similar, specific fluorescent signal curves and positive outcomes (Figure 8). This indicates that the RAA–CRISPR/Cas13a assay has good detection stability for TGEV.

### 3.9. Clinical Sample Detection of RAA–CRISPR/Cas13a Assay

A total of 140 clinical samples were tested using both the RAA–CRISPR/Cas13a assay and the RT–qPCR and RT–PCR assays to evaluate the clinical application of the method. The comparative analysis of the test results is shown in Table 7. Both methods produced the same results for all clinical samples, indicating a 100% coincidence rate. Among them, a total of eight positive samples were detected with a positive rate of 5.7%, and the test results were completely consistent between the two detection methods.

## 4. Discussion

TGE is a widely distributed, difficult–to–prevent, and difficult–to –control intestinal infectious disease that causes severe diarrhea in swine. Many countries and regions have reported outbreaks of TGE in swine herds, a condition that significantly impacts the gastrointestinal health of pigs and the development of the pig industry. Additionally, sick pigs are often co–infected and may harbor other diarrheal pathogens [22]. Notably, infected pigs with PRCV can experience mild TGEV onset at any age, which may obscure the presence of TGEV infection [6]. Early detection of TGEV is essential for implementing effective prevention and control measures. Reliable viral nucleic acid detection methods can offer accurate and efficient technical support for virus detection [23]. Therefore, in this study, we developed a novel TGEV nucleic acid assay based on RAA–CRISPR/Cas13a, focusing on the TGEV S gene.

Existing studies have shown that the CRISPR/Cas13a system can tolerate at most one mismatch between crRNA and target RNA [11,24]. Cas13a nuclease alone requires several hours to achieve amol sensitivity, necessitating a nucleic acid target pre–amplification step to increase the concentration for the CRISPR/Cas13a assay. RAA, which departs from traditional PCR techniques, can rapidly amplify nucleic acid targets in a short time. However, previous studies have indicated that RAA primers and probes can tolerate mismatches of up to nine bases with the target sequence [25], which significantly increases the risk of non–specific amplification due to these tolerable multi–base mismatches [26].

In view of the respective shortcomings of the two methods described above, we established the RAA–CRISPR/Cas13a assay. We began by designing and screening specific RAA primers and corresponding crRNA based on the conserved sequence of the TGEV S gene to avoid interference from PRCV nucleic acid. Given the limited number of Cas13a proteins and the potential for abundant crRNA to compete with the ssRNA reporter for cleavage [27,28,29], we further optimized the RAA–CRISPR/Cas13a assay through orthogonal experiments. This optimization focused on three factors: primer concentration, which can affect the amplification efficiency of RAA, and crRNA concentration and ssRNA reporter concentration, both of which can affect the cleavage efficiency of the Cas13a protease in the reaction system.

The optimized RAA–CRISPR/Cas13a assay represents a significant advancement over LAMP methods for TGEV detection. Although LAMP has been widely adopted for its field adaptability and simple equipment requirements, the CRISPR–based system demonstrates superior performance, with higher sensitivity (4.13 copies/µL vs. LAMP’s typical 10–100 copies/µL), specificity through RNA–guided target recognition, and a faster turnaround time (40 min). The closed–tube design of the RAA–CRISPR/Cas13a assay additionally minimizes contamination risks that can affect open–tube LAMP reactions [30,31,32]. Current CRISPR implementation faces challenges in field deployment due to fluorescence detection requirements and higher reagent costs, while its exceptional detection capabilities make it particularly valuable for laboratory–based confirmatory testing and settings where maximum accuracy is paramount. The field application of the RAA–CRISPR/Cas13a assay is expected to be realized using portable fluorescence detectors or lateral flow chromatographic test strips, offering more efficient and practical solutions for the clinical detection of TGEV [33,34].

## Figures and Tables

**Table 1:** Information on recombinase–aided amplification (RAA) primers.

Number	Primer Sequences	Primer Pairing	Product Size, bp
F1	5′-GGTGTTAGGTGATTATTTTCCTACTGTACA-3′	F1/R1	308
F2	5′-ACGGTTAAACGTAGTCGTTAATGGATACCC-3′	F2/R1	164
F3	5′-CGGTTAAACGTAGTCGTTAATGGATACCCA-3′	F3/R1	163
R1	5′-ACCTGCACTCACTACCCCAATTGCAAGTCA-3′	F1/R2	309
R2	5′-AACCTGCACTCACTACCCCAATTGCAAGTC-3′	F2/R2	165
		F3/ R2	164

**Table 2:** L9 (3^3^) orthogonal analysis protocol design for the detection method based on RAA–CRISPR/Cas13a.

Test No.	Experimental Condition Parameters					
	A (Reporter RNA Concentration, μM)		B (Primer Concentration, μM)		C (crRNA Concentration, μM)	
1	1	8	1	8	1	2.5
2	1	8	2	10	3	10
3	1	8	3	12	2	5
4	2	10	1	8	3	10
5	2	10	2	10	2	5
6	2	10	3	12	1	2.5
7	3	12	1	8	2	5
8	3	12	2	10	1	2.5
9	3	12	3	12	3	10

**Table 3:** Repeatability test results of qPCR.

Intra–Repetitive Assay			Inter–Repetitive Assay		
$\mathrm{Average}\;\mathrm{Value}\;(\overset{–}{x})$	Standard Deviation (s)	Coefficient of Variation (CV/%)	$\mathrm{Average}\;\mathrm{Value}\;(\overset{–}{x})$	Standard Deviation (s)	Coefficient of Variation (CV/%)
35.58	0.44	1.2	36.01	0.52	1.4
35.86	0.41	1.1			
36.60	0.42	1.1			

**Table 4:** Results of L9 (3^3^) orthogonal tests.

Test No.	Experimental Condition Parameters			
	A (Probe)	B (Primer)	C (crRNA)	Ct Value
1	1	1	1	2.89
2	1	2	3	2.49
3	1	3	2	2.56
4	2	1	3	1.84
5	2	2	2	1.98
6	2	3	1	2.01
7	3	1	2	1.88
8	3	2	1	1.84
9	3	3	3	1.88

**Table 5:** Analysis of L9 (3^3^) orthogonal test results.

	Results of Orthogonal Test		
	A (Probe)	B (Primer)	C (crRNA)
K1	7.93325	6.60910	6.74525
K2	5.83047	6.31189	6.41953
K3	5.60377	6.44651	6.20272
k1	2.64442	2.20303	2.24842
k2	1.94349	2.10396	2.13984
k3	1.86792	2.14884	2.06757
R	0.77649	0.09907	0.18084
Optimal conditions	A3	B2	C3

**Table 6:** Three–factor ANOVA results.

Source of Variation	*SS*	*df*	*MS*	*F*	*p*
Intercept	41.689	1	41.689	1801.233	0.001 **
A (ssRNA reporter concentration, μM)	1.109	2	0.554	23.957	0.040 *
B (primer concentration, μM)	0.015	2	0.008	0.325	0.755
C (crRNA concentration, μM)	0.047	2	0.024	1.026	0.494
Residual	0.046	2	0.023		

Note: R^2^ = 0.962. * *p* < 0.05; ** *p* < 0.01.

**Table 7:** Comparison of test results for clinical samples obtained using the RAA–CRISPR/Cas13a assay, RT–qPCR method, and RT–PCR method.

No.	Region	Time	Quantity	Methods		
				RAA–CRISPR/Cas13a Detection Positive Results	RT–qPCR Detection Positive Results	RT–PCR Detection Positive Results
1	Wanzhou	19 February 2023	70	5/70	5/70	5/70
2	Luzhou	09 March 2023	4	1/4	1/4	1/4
3	Wulong	04 November 2023	20	2/20	2/20	2/20
4	Changshou	02 December 2023	8	0/8	0/8	0/8
5	Banan	17 December 2023	11	0/11	0/11	0/11
6	Rongchang	23 February 2024	16	0/16	0/16	0/16
7	Dazhou	21 November 2024	7	0/7	0/7	0/7
8	Longchang	24 December 2024	4	0/4	0/4	0/4

## Data Availability

The raw datasets used and/or analyzed in the current study are available from the corresponding authors upon request.

## Abbreviations

The following abbreviations are used in this manuscript:

ANOVA	Analysis of variance
ASFV	African swine fever virus
CRISPR	Clustered regularly interspaced short palindromic repeat
Cas	CRISPR–associated proteins
CV	Coefficient of variation
CSFV	Classical swine fever virus
PCV2	Porcine circovirus 2
PDCoV	Porcine delta coronavirus
PEDV	Porcine epidemic diarrhea virus
PKoV	Porcine kobuvirus
PRCV	Porcine respiratory coronavirus
PRRSV	Porcine reproductive and respiratory syndrome virus
SARS-CoV-2	Severe acute respiratory syndrome coronavirus 2
TGEV	Porcine transmissible gastroenteritis virus
RAA	Recombinase–aided amplification
